# Draft Genome Sequence of *Lactococcus lactis* subsp. *lactis* Strain YF11

**DOI:** 10.1128/genomeA.00599-13

**Published:** 2013-08-08

**Authors:** Yuhui Du, Lifu Song, Wenjing Feng, Guangsheng Pei, Ping Zheng, Zhichao Yu, Jibin Sun, Jianjun Qiao

**Affiliations:** Department of Pharmaceutical Engineering, School of Chemical Engineering and Technology, Tianjin University, Tianjin, Chinaa; Tianjin Institute of Industrial Biotechnology, Chinese Academy of Sciences, Tianjin, Chinac; Key Laboratory of Systems Bioengineering, Ministry of Education, Tianjin, Chinab; Key Laboratory of Systems Microbial Biotechnology, Chinese Academy of Sciences, Tianjin, Chinad

## Abstract

*Lactococcus lactis* subsp. *lactis* strain YF11 is a food preservative bacterium with a high capacity to produce nisin. Here, we announce the draft genome sequence of *Lactococcus lactis* subsp. *lactis* YF11 (2,527,433 bp with a G+C content of 34.81%).

## GENOME ANNOUNCEMENT

*Lactococcus lactis* is a mesophilic, Gram-positive bacterium that ferments hexose to lactic acid. It is a long tradition to use this bacterium in the production of fermented foods and beverages. This species also performs a vital role in the production of a natural preservative, nisin. At present, only a few *Lactococcus* strains, like *Lactococcus lactis* subsp. *lactis* strain CV56 and *Lactococcus lactis* subsp. *lactis* strain IO-1, have been found to secrete nisin (1, 2). *Lactococcus lactis* subsp. *lactis* YF11 is a new strain that produces nisin efficiently, and its mutant is successfully used in the industrial production of nisin in China. *L. lactis* YF11 is accessible from the China General Microbiological Culture Collection Center under the accession number CGMCC7.52.

Nisin, a lantibiotic, is an excellent food preservative because of its high antibacterial activity and low toxicity for humans (3, 4, 5, 6). Nisin inhibits virtually all Gram-positive bacteria, such as food-borne pathogens and spoilage microorganisms, and also acts on several Gram-negative bacteria (7, 8). Nisin can be degraded into amino acids by proteases in the human digestive system. For its efficiency, safety, and nonaccumulation, nisin is broadly applied as a food biopreservative (9–12).

The natural strain *L. lactis* YF11 without any modification can produce nisin amounts as high as 1,025 IU/ml, and its tolerance to nisin is about 5,000 IU/ml. The release of the genome sequence of *L. lactis* YF11 will help us investigate the nisin production mechanism with regard to nisin biosynthesis as well as nisin tolerance and eventually will facilitate the rational improvement of the strain by metabolic engineering.

The total genomic DNA of *L. lactis* YF11 was purified with the DNeasy blood and tissue kit (Qiagen). Two libraries, containing 300 bp and 400 bp, respectively, were constructed. Deep sequencing was performed with the Illumina Hiseq 2000 system applying the paired-end strategy of a 100-bp reading length. More than 10 Gbp was generated, representing 1,800-fold coverage of the genome. The reads were assembled into contigs by the genome assembler software Velvet (13) with the help of the reference genome of *Lactococcus lactis* subsp. *lactis* strain IL1403 (GenBank accession number AE005176). Open reading frames (ORFs) were identified by Glimmer version 3.02 (14). Annotation was done by BLASTP against UniRef90 and the KEGG database.

The draft genome sequence of *L. lactis* YF11 contains 71 contigs covering 2,527,433 bp, with a G+C content of 34.81%, and 2,529 protein-coding sequences (CDS) were annotated. All the genes responsible for the complex biosynthesis of nisin were found in the genome, including the structural gene (*nisZ*); genes involved in posttranslational modifications (*nisB* and *nisC*), transportation (*nisT*), and extracellular precursor processing (*nisP*); genes encoding immunity (*nisI* and *nisFEG*); and regulatory genes (*nisR* and *nisK*). KAAS (the Kegg automatic annotation server) was also used to construct a draft metabolic network (15) containing 142 metabolic pathways. Further analysis will provide significant guides for understanding the hyperproduction mechanism of nisin and for strain improvement.

### Nucleotide sequence accession number.

This whole-genome shotgun project has been deposited at DDBJ/EMBL/GenBank under the accession number APAV00000000.
